# The Risk Ratio of Immune-Related Colitis, Hepatitis, and Pancreatitis in Patients With Solid Tumors Caused by PD-1/PD-L1 Inhibitors: A Systematic Review and Meta-Analysis

**DOI:** 10.3389/fonc.2020.00261

**Published:** 2020-02-28

**Authors:** Yuan Tian, Zewen Zhang, Xiaowei Yang, Donghua Li, Li Zhang, Zhuoqi Li, Shuisheng Zhang, Yantao Mao, Chenxing Jin, Yi Zhao

**Affiliations:** ^1^Department of Radiotherapy Oncology, Shandong Provincial Qianfoshan Hospital, The First Hospital Affiliated With Shandong First Medical University, Jinan, China; ^2^Department of Imaging Medicine and Nuclear Medicine, Qilu Medical College, Shandong University, Jinan, China; ^3^Department of Hepatobiliary Intervention, School of Clinical Medicine, Beijing Tsinghua Changgung Hospital, Tsinghua University, Beijing, China; ^4^Department of Radiotherapy, The People's Hospital of Yuncheng County, Heze, China; ^5^Department of Pathology, Zaozhuang Municipal Hospital, Zaozhuang, China; ^6^Key Laboratory of Carcinogenesis and Translational Research (Ministry of Education/Beijing), Division of Etiology, Peking University Cancer Hospital and Institute, Beijing, China; ^7^Department of General Surgery, Peking University Third Hospital, Beijing, China; ^8^Department of Oncology, Yantaishan Hospital of Shandong Province, Yantai, China; ^9^Department of Oncology, The First Affiliated Hospital of Dalian Medical University, Dalian, China

**Keywords:** PD-1/PD-L1, chemotherapy, immune-related inflammation, solid tumor, meta-analysis

## Abstract

**Purpose:** The meta-analysis was put into practice in evaluating the risk ratio of immune-related digestive system inflammation in patients with solid tumors caused by PD-1/PD-L1 inhibitors.

**Method:** The process of the meta-analysis was performed by us according to the Preferred Reporting Items for Systematic Reviews and Meta-analyses (PRISMA) guidelines.

**Results:** After screening and eligibility assessment, a total of 26 clinical trials involving 16,409 patients were selected for the final quantitative synthesis. Immune-related digestive system inflammations, including colitis, hepatitis, pancreatitis, were evaluated separately. Compared with chemotherapy, PD-1/PD-L1 inhibitors led to an increase in the incidence risk of all grade colitis (RR = 2.43, 95% CI: [1.23, 4.82], *P* = 0.01). Similar incidence trend could also be seen when PD-1/PD-L1 inhibitors were combined with chemotherapy (RR = 2.62, 95% CI: [1.25, 5.48], *P* = 0.01). Whether compared with Nivolumab plus Ipilimumab or Ipilimumab alone, the incidence risk of colitis in the Nivolumab group was significantly lower than that of the control group. Similar analysis results could also be seen in the incidence risk of hepatitis. We did not find a statistically significant effect on the incidence of immune-related pancreatitis after the use of PD-1/PD-L1 inhibitors.

**Conclusion:** The use of PD-1/PD-L1 inhibitors increased the incidence risk of immune-related colitis and hepatitis, but this potential to increase the incidence risk of the disease was weaker than Ipilimumab.

## Introduction

Immune-related digestive system inflammation, including colitis, hepatitis, pancreatitis, can be caused by a variety of pathogenic factors, such as genetic abnormality, autoimmune factors, immune-related drugs, viral infections, and so on (1–4). PD-L1(B7-H1) is thought to be involved in the regulation of cellular and humoral immune responses through the PD-1 receptor on activated T and B cells (5, 6). PD-1/PD-L1 inhibitors block the negative regulatory signal by inhibiting the binding of PD-1 and PD-L1, allowing T cells to restore their activity, enhancing immune response, and thereby exerting anti-tumor activity (7–10). Satisfactory anti-tumor efficacy had been shown in plenty of clinical trials (11–36). However, with the increasing clinical application in different kinds of malignant diseases, more and more PD-1/PD-L1 related side toxicity effects had been reported, and immune-related digestive system inflammation was one of them (11–36).

Although the incidence rate of immune-related digestive system inflammation was not as high as myelosuppression, it had an important impact on the quality of patients lives and even the survival prognosis (11–36). Some of them, such as pancreatitis, might even jeopardize the life of patient if it was neglected in the process of therapy (13, 15–17, 20, 22, 26, 27, 30). Therefore, we should pay enough attention to immune-related gastrointestinal inflammation in clinical work. However, due to the interference from other anti-tumor drugs, we were unable to clearly define the relationship between immune related digestive system inflammation and PD-1/PD-L1 inhibitors (15–18, 36). Furthermore, the combination of PD-1/PD-L1 inhibitors with other anti-tumor immunoassays, such as Ipilimumab, also increased the difficulty for our judgment (13, 24, 29, 35).

To investigate the relationship between incidence risk of immune-related digestive system inflammation and PD-1/PD-L1 inhibitors, we performed this meta-analysis.

## Methods

The meta-analysis was conducted according to the Preferred Reporting Items for Systematic Reviews and Meta-analyses (PRISMA) statement (37).

### Search Strategy

The PubMed website was used to identify clinical trials involving PD-1/PD-L1 inhibitors for solid tumor patients. Relevant randomized clinical trials (RCTs), reported from inception to July 31, 2019, were collected by using search keywords and Medical Subject Headings (MeSH) terms pertinent to the intervention of interest, such as cancer, tumor, PD-1/PD-L1, nivolumab, opdivo, pembrolizumab, immune checkpoint inhibitor, Keytruda, Imfinzi, MK-3475, atezolizumab, Tecentriq, MPDL3280A, avelumab, Bavencio, durvalumab, and BMS-963558. Furthermore, some RCTs that could not be found on the PubMed website were obtained by searching and checking references of other systematic reviews, meta-analyses, and conference proceedings of the American Society of Clinical Oncology, the European Society for Medical Oncology, the American Association for Cancer Research, and the World Conference on Cancers.

According to our analysis design, the inclusion criteria were as follows: (1) RCTs would be preferred choices, (2) PD-1/PD-L1 inhibitors were used as an anti-tumor therapy, (3) The treatment regimen of the control group included anti-tumor drugs or placebo, (4) All enrolled patients were diagnosed with solid tumors rather than hematological malignancies, (5) At least one of Immune-related Digestive System Inflammation (colitis, hepatitis, and pancreatitis) was reported, (6) The results of the clinical trials are reported in English or reported in other languages and English.

### Data Extraction

Four investigators (Zewen Zhang, Xiaowei Yang, Donghua Li, Li Zhang) were designated to determine the eligibility and duplicate independently by checking titles and abstracts of enrolled studies. The data categories of enrolled studies were collected as follows: first author, the year of publication, study name and number, treatment regimen, number of evaluable cases, related drug name, phase stage, tumor type, incidence rate of immune-related digestive system inflammation.

Both all-grade and grade 3–5 immune-related digestive system inflammation were taken into account for the final comprehensive meta-analysis. The basic characteristics of all enrolled studies would be summarized in Table 1.

**Table 1:** Basic characteristics of the included studies.

**No**.	**References**	**Study name (NCT number)**	**Drug name (PD-1/PD-L1)**	**Treatment regimen**	**Number of evaluable patients**	**Previous therapy**	**Phase**	**Randomized controlled trial (RCT)**	**Tumor type**
1	Rini et al. (11)	IMmotion151 (NCT02420821)	Atezolizumab (PD-L1)	Atezolizumab + Bevacizumab vs. Sunitinib	897	No	III	Yes	Renal Cell Carcinoma
2	Mok et al. (12)	KEYNOTE-042 (NCT02220894)	Pembrolizumab (PD-1)	Pembrolizumab vs. Chemotherapy	1,241	No	III	Yes	NSCLC
3	Hodi et al. (13)	CheckMate 067 (NCT01844505)	Nivolumab (PD-1)	Nivolumab + Ipilimumab vs. Nivolumab or Ipilimumab	937	No	III	Yes	Advanced Melanoma
4	Cohen et al. (14)	KEYNOTE-040 (NCT02252042)	Pembrolizumab (PD-1)	Pembrolizumab vs. (Methotrexate, Docetaxel, or Cetuximab)	480	Yes	III	Yes	Head-and-neck Squamous Cell Carcinoma
5	Schmid et al. (15)	IMpassion130 (NCT02425891)	Atezolizumab (PD-L1)	Atezolizumab + Nab-paclitaxel vs. Placeo + Nab-paclitaxel	890	No	III	Yes	Advanced Triple-Negative Breast Cancer
6	Horn et al. (16)	IMpower133 (NCT02763579)	Atezolizumab (PD-L1)	Atezolizumab + Carboplatin + Etoposide vs. Placebo + Carboplatin + Etoposide	394	No	III	Yes	SCLC
7	Socinski et al. (17)	IMpower150 (NCT02366143)	Atezolizumab (PD-L1)	Atezolizumab + BCP vs. Placeo + BCP	787	No	III	Yes	Metastatic non-squamous NSCLC
8	Paz-Ares et al. (18)	KEYNOTE-407 (NCT02775435)	Pembrolizumab (PD-1)	Pembrolizumab + Carboplatin + Paclitaxel vs. Placebo + Carboplatin + Paclitaxel	558	No	III	Yes	Metastatic squamous NSCLC
9	Barlesi et al. (19)	JAVELIN Lung 200 (NCT02395172)	Avelumab (PD-L1)	Avelumab vs. Docetaxel	792	Yes	III	Yes	Advanced NSCLC
10	Shitara et al. (20)	KEYNOTE-061 (NCT02370498)	Pembrolizumab (PD-1)	Pembrolizumab vs. Paclitaxel	570	Yes	III	Yes	Advanced Gastric or gastro-esophageal Junction Cancer
11	Hida et al. (21)	NCT02008227	Atezolizumab (PD-L1)	Atezolizumab vs. Docetaxel	101	Yes	III	Yes	Locally Advanced/Metastatic NSCLC
12	Eggermont et al. (22)	NCT02362594	Pembrolizumab (PD-1)	Pembrolizumab vs. Placebo	1,009	No	III	Yes	Completely resected stage III Melanoma
13	Kang et al. (23)	ONO-4538-12, ATTRACTION-2 (NCT02267343)	Nivolumab (PD-1)	Nivolumab vs. Placebo	491	Yes	III	Yes	Advanced Gastric or Gastro-esophageal Junction Cancer
14	Wolchok et al. (24)	CheckMate 067 (NCT01844505)	Nivolumab (PD-1)	Nivolumab vs. Ipilimumab or Nivolumab + Ipilimumab	937	No	III	Yes	Advanced Melanoma
15	Schachter et al. (25)	KEYNOTE-006 (NCT01866319)	Pembrolizumab (PD-1)	Pembrolizumab vs. Ipilimumab	811	No	III	Yes	Advanced Melanoma
16	Bellmunt et al. (26)	KEYNOTE-045 (NCT02256436)	Pembrolizumab (PD-1)	Pembrolizumab vs. Chemotherapy	521	Yes	III	Yes	Advanced Urothelial Carcinoma
17	Reck et al. (27)	KEYNOTE-024 (NCT02142738)	Pembrolizumab (PD-1)	Pembrolizumab vs. Chemotherapy	304	No	III	Yes	PD-L1-Positive NSCLC
18	Ferris et al. (28)	CheckMate 141 (NCT02105636)	Nivolumab (PD-1)	Nivolumab vs. (Methotrexate, Docetaxel, or Cetuximab)	347	Yes	III	Yes	Recurrent Squamous-Cell Carcinoma of the Head and Neck
19	Antonia et al. (29)	CheckMate 032 (NCT01928394)	Nivolumab (PD-1)	Nivolumab vs. Nivolumab + Ipilimumab	213	Yes	I/II	N/A	Recurrent SCLC
20	Herbst et al. (30)	KEYNOTE-010 (NCT01905657)	Pembrolizumab (PD-1)	Pembrolizumab vs. Docetaxel	991	Yes	II/III	Yes	Advanced NSCLC
21	Hodi et al. (31)	CheckMate 069 (NCT01927419)	Nivolumab (PD-1)	Nivolumab + Ipilimumab vs. Ipilimumab	140	No	II	Yes	Advanced Melanoma
22	Borghaei et al. (32)	CheckMate 057 (NCT01673867)	Nivolumab (PD-1)	Nivolumab vs. Docetaxel	555	Yes	III	Yes	Non-squamous NSCLC
23	Brahmer et al. (33)	CheckMate 017 (NCT01642004)	Nivolumab (PD-1)	Nivolumab vs. Docetaxel	260	Yes	III	Yes	Squamous NSCLC
24	Weber et al. (34)	CheckMate 037 (NCT01721746)	Nivolumab (PD-1)	Nivolumab vs. Chemotherapy	631	Yes	III	Yes	Advanced Melanoma
25	Larkin et al. (35)	CheckMate 067 (NCT01844505)	Nivolumab (PD-1)	Nivolumab and Ipilimumab vs. Nivolumab or Ipilimumab	945	No	III	Yes	Unresectable stage III or IV Melanoma
26	Gandhi et al. (36)	KEYNOTE-189 (NCT02578680)	Pembrolizumab (PD-1)	Pembrolizumab + chemotherapy vs. Placebo + Chemotherapy	607	No	III	Yes	NSCLC

*RCT, Randomized controlled trial; BCP, Bevacizumab plus Carboplatin plus Paclitaxel; NSCLC, Non-Small Cell Lung Cancer; PD-1, Programmed Cell Death 1; PD-L1, Programmed Cell Death Ligand 1; SCLC, Small Cell Lung Cancer; CP, Carboplatin plus Paclitaxel; PC, Pemetrexed plus a platinum-based drug; Chemotherapy, Carboplatin plus Pemetrexed, Cisplatin plus Pemetrexed, Carboplatin plus Gemcitabine, Cisplatin plus Gemcitabine, or Carboplatin plus Paclitaxel; N/A, No Available*.

### Statistical Analysis

Risk Ratio (RR) was used to assess the risk of developing immunological gastrointestinal inflammation. 95% confidence interval (CI) were calculated by random effect (RE). *P* < 0.05 was regarded as statistically significance. Statistical tests were all two-sided. Subgroup analysis would be performed according to the type of tumor, treatment plan, and specific drug name. Cochrane's Q and the *I*^2^ statistic, proposed by Higgins and colleagues, were used for checking the heterogeneity among enrolled trials (37, 38). *I*^2^ values <25, 25–50, and >50% indicated low, medium and high heterogeneity, respectively. Newcastle-Ottawa scale, Funnel plot and Egger's test were used for assessing the bias of the analysis result (37, 39–42). Four investigators (Zewen Zhang, Xiaowei Yang, Donghua Li, Li Zhang) were appointed to evaluate the quality of all enrolled trials.

The evaluation indicators related to the quality of the included trials, named Newcastle-Ottawa scale, including random sequence generation, allocation concealment, blinding of participants and personnel, blinding of outcome assessment, incomplete outcome data, and selective outcome reporting, would be checked one by one and summarized in a figure (42). Review Manager 5.3, proposed by the Cochrane Collaboration, was used for the final comprehensive analysis.

## Results

Twenty-six clinical trials related publications involving 16,409 patients were collected for the final comprehensive meta-analysis according to our enrolled criteria. The PRISMA Flow Diagram was displayed in Supplemental Figure 7, while the evaluation results of bias was shown in Supplemental Figure 8 (42). The basic information and clinical characteristics were provided in Table 1 (11–36). Nivolumab (*n* = 10), Pembrolizumab (*n* = 10), Atezolizumab (*n* = 5), and Avelumab (*n* = 1), listed in Table 1, were used in corresponding clinical trials (11–36). Eight kind of tumors, including renal cell carcinoma (*n* = 1), NSCLC (*n* = 10), melanoma (*n* = 7), head-and-neck carcinoma (*n* = 2), triple-negative breast cancer (*n* = 1), SCLC (*n* = 2), gastric or gastro-esophageal junction cancer (*n* = 2), urothelial carcinoma (*n* = 1), were referred. Among these enrolled clinical trials, there were 1 phase I/II clinical trial, 1 phase II clinical trial, 1 phase II/III clinical trial, and 23 phase III clinical trials. Twenty-five clinical trials were reported to be randomized controlled trial (RCT), while the RCT information of 1 clinical trial was unavailable (29). Previous treatments could be found in 12 trials (14, 19–21, 23, 26, 28–30, 32–34). Publication bias, checked by Harbord's test (37), was shown in the form of funnel plots (Supplemental Figures 1–6).

### Incidence Risk of Colitis

**Figure 1:**
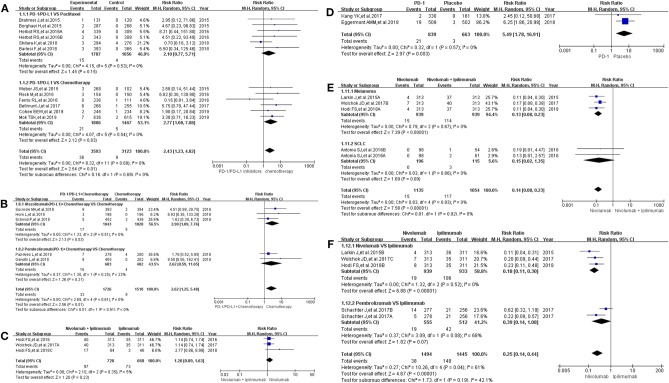
Forest plots for the risk ratio of colitis for all grade. **(A)** Forest plots for the risk ratio of treatment related colitis (PD-1/PD-L1 vs. Docetaxel/Paclitaxel or Chemotherapy). Subgroup analysis was performed according to the composition of chemotherapy in the control group. **(B)** Forest plots for the risk ratio of treatment related colitis (PD-1/PD-L1 + Chemotherapy vs. Chemotherapy). Subgroup analysis was performed based on the drug type (PD-1 or PD-L1) of the experimental group. **(C)** Forest plots for the risk ratio of treatment related colitis (Nivolumab + Ipilimumab vs. Ipilimumab). **(D)** Forest plots for the risk ratio of treatment related colitis (PD-1 vs. Placebo). **(E)** Forest plots for the risk ratio of treatment related colitis (Nivolumab vs. Nivolumab + Ipilimumab). Subgroup analysis was performed according to the tumor type. **(F)** Forest plots for the risk ratio of treatment related colitis (Nivolumab vs. Ipilimumab).

All grade of colitis was evaluated first (11–20, 22–36). Clinical trials included in the study were divided into 7 groups according to treatment methods for the final comprehensive analysis (Figure 1). Compared with chemotherapy, PD-1/PD-L1 inhibitors led to an increase in the incidence risk of colitis (RR = 2.43, 95% CI: [1.23, 4.82], *I*^2^ = 0%, *Z* = 2.54 (*P* = 0.01); Figure 1A), especially in clinical trials related with combined chemotherapy (RR = 2.77, 95% CI: [1.08, 7.08], *I*^2^ = 0%, *Z* = 2.12 (*P* = 0.03); Figure 1A) (12, 14, 19, 20, 26–28, 30, 32–34). Similar incidence trend could also be seen when PD-1/PD-L1 inhibitors were combined with chemotherapy (RR = 2.62, 95% CI: [1.25, 5.48], *I*^2^ = 0%, *Z* = 2.56 (*P* = 0.01); Figure 1B) (15–18, 36). No statistical significance could be found when Nivolumab combined with Ipilimumab were compared with Ipilimumab alone (RR = 1.20, 95% CI: [0.89, 1.63], *I*^2^ = 5%, *Z* = 1.20 (*P* = 0.23); Figure 1C) (13, 24, 31). There was an obvious higher incidence risk of all grade colitis when PD-1 inhibitors were compared with placebo (RR = 5.49, 95% CI: [1.78, 16.91], *I*^2^ = 0%, *Z* = 2.97 (*P* = 0.003); Figure 1D) (22, 23). Opposite incidence trend was seen in another two groups (Figures 1E,F). Whether compared with Nivolumab plus Ipilimumab or Ipilimumab alone, the incidence risk of colitis in the Nivolumab group was significantly lower than that of the control group, and the difference was statistically significant (RR = 0.15, Figure 1E; RR = 0.25, Figure 1F) (13, 24, 25, 29, 35). Meta-analysis was not performed in group G because only one group of clinical trial was enrolled (11). The corresponding funnel plots of RR were gathered in Supplemental Figure 1. Obvious heterogeneity was only found in Figure 1F (*I*^2^ = 61%).

**Figure 2:**
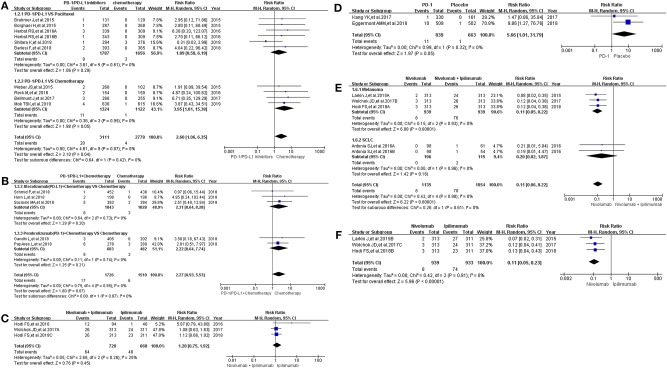
Forest plots for the risk ratio of colitis for grade 3–5. **(A)** Forest plots for the risk ratio of treatment related colitis (PD-1/PD-L1 vs. Docetaxel/Paclitaxel or Chemotherapy). Subgroup analysis was performed according to the composition of chemotherapy in the control group. **(B)** Forest plots for the risk ratio of treatment related colitis (PD-1/PD-L1 + Chemotherapy vs. Chemotherapy). Subgroup analysis was performed based on the drug type (PD-1 or PD-L1) of the experimental group. **(C)** Forest plots for the risk ratio of treatment related colitis (Nivolumab + Ipilimumab vs. Ipilimumab). **(D)** Forest plots for the risk ratio of treatment related colitis (PD-1 vs. Placebo). **(E)** Forest plots for the risk ratio of treatment related colitis (Nivolumab vs. Nivolumab + Ipilimumab). Subgroup analysis was performed according to the tumor type. **(F)** Forest plots for the risk ratio of treatment related colitis (Nivolumab vs. Ipilimumab).

Then, we took the same method to deal with all the data for evaluating the incidence risk of grade 3–5 colitis (11–13, 15–20, 22–24, 26, 27, 29–36). However, the analysis results of statistical significance could be seen only in Figures 2E,F (13, 24, 29, 35), while no statistical significant results were seen in Figures 2A–D (12, 13, 15–20, 22–24, 26, 27, 30–34, 36). Compared with Nivolumab plus Ipilimumab, the incidence risk of colitis in the Nivolumab group was sificantly lower than that of the control group (RR = 0.11, 95% CI: [0.06, 0.22], *I*^2^ = 0%, *Z* = 6.22 (*P* < 0.00001); Figure 2E), especially in the subgroup of melanoma (RR = 0.11, 95% CI: [0.05, 0.22], *I*^2^ = 0%, *Z* = 6.08 (*P* < 0.00001); Figure 2E) (13, 24, 29, 35). Similar analysis results were shown in Figure 2F when Nivolumab was compared with Ipilimumab [RR = 0.11, 95% CI: [0.05, 0.23], *I*^2^ = 0%, *Z* = 5.96 (*P* < 0.00001)] (13, 24, 35). The corresponding funnel plots of RR were gathered in Supplemental Figure 2. No obvious heterogeneity was found among all groups (*I*^2^ = 0%).

### Incidence Risk of Hepatitis

**Figure 3:**
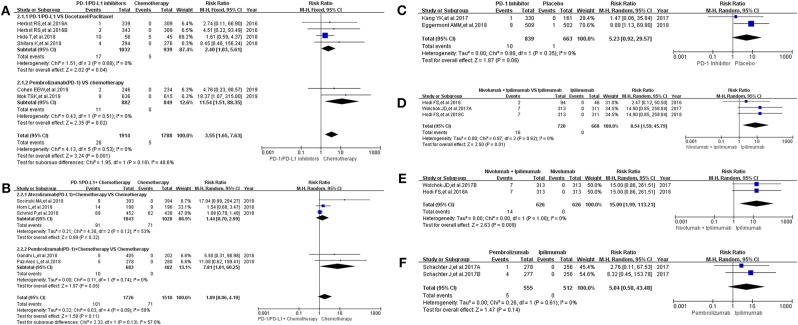
Forest plots for the risk ratio of hepatitis for all grade. **(A)** Forest plots for the risk ratio of treatment related hepatitis (PD-1/PD-L1 vs. Docetaxel/Paclitaxel or Chemotherapy). Subgroup analysis was performed according to the composition of chemotherapy in the control group. **(B)** Forest plots for the risk ratio of treatment related hepatitis (PD-1/PD-L1 + Chemotherapy vs. Chemotherapy). Subgroup analysis was performed based on the drug type (PD-1 or PD-L1) of the experimental group. **(C)** Forest plots for the risk ratio of treatment related hepatitis (PD-1 vs. Placebo). **(D)** Forest plots for the risk ratio of treatment related hepatitis (Nivolumab + Ipilimumab vs. Ipilimumab). **(E)** Forest plots for the risk ratio of treatment related hepatitis (Nivolumab + Ipilimumab vs. Nivolumab). **(F)** Forest plots for the risk ratio of treatment related hepatitis (Nivolumab vs. Ipilimumab).

Sixteen clinical trials with the information of all grade hepatitis were taken into account for meta-analysis (12–18, 20–25, 30, 31, 36). They were divided into seven groups according to treatment methods for the final comprehensive analysis (Figure 3). Compared with chemotherapy, the incidence risk of all grade hepatitis was higher in PD-1/PD-L1 inhibitors group (RR = 3.55, 95% CI: [1.65, 7.63], *I*^2^ = 0%, *Z* = 3.24 (*P* = 0.001); Figure 3A) (12, 14, 20, 21, 30), especially in the subgroup of Pembrolizumab compared with combined chemotherapy (RR = 11.54, 95% CI: [1.51, 88.35], *I*^2^ = 0%, *Z* = 2.35 (*P* = 0.02); Figure 3A) (12, 14). However, when PD-1/PD-L1 inhibitors plus chemotherapy was compared with chemotherapy, the analysis result of incidence risk was considered to be no significant (RR = 1.89, 95% CI: [0.86, 4.19], *I*^2^ = 50%, *Z* = 1.58 (*P* = 0.11); Figure 3B) (15–18, 36), even if in each subgroup. Similar incidence trend could also be seen when PD-1 inhibitor was compared with placebo or Ipilimumab (Figures 3C,F) (22, 23, 25). There was an obvious higher incidence risk of all grade hepatitis when Nivolumab plus Ipilimumab were compared with Ipilimumab (RR = 8.54, 95% CI: [1.59, 45.79], *I*^2^ = 0%, *Z* = 2.50 (*P* = 0.01); Figure 3D) (13, 24, 31). When the control group was Nivolumab, similar result was seen in Figure 3E [RR = 15.00, 95% CI: [1.99, 113.21], *I*^2^ = 0%, *Z* = 2.63 (*P* = 0.009)] (13, 24). The corresponding funnel plots of RR were summarized in Supplemental Figure 3. Moderate heterogeneity was only found in Figure 3B (*I*^2^ = 50%).

**Figure 4:**
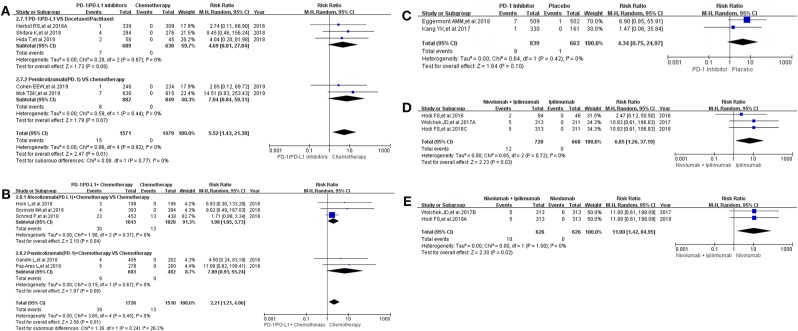
Forest plots for the risk ratio of hepatitis for grade 3–5. **(A)** Forest plots for the risk ratio of treatment related hepatitis (PD-1/PD-L1 vs. Docetaxel/Paclitaxel or Chemotherapy). Subgroup analysis was performed according to the composition of chemotherapy in the control group. **(B)** Forest plots for the risk ratio of treatment related hepatitis (PD-1/PD-L1 + Chemotherapy vs. Chemotherapy). Subgroup analysis was performed based on the drug type (PD-1 or PD-L1) of the experimental group. **(C)** Forest plots for the risk ratio of treatment related hepatitis (PD-1 vs. Placebo). **(D)** Forest plots for the risk ratio of treatment related hepatitis (Nivolumab + Ipilimumab vs. Ipilimumab). **(E)** Forest plots for the risk ratio of treatment related hepatitis (Nivolumab + Ipilimumab vs. Nivolumab).

The same grouping and subgroup approach as before were taken for evaluating the incidence risk of grade 3–5 hepatitis. Fourteen clinical trials with the information of hepatitis were taken into account for the final meta-analysis (12–18, 20–24, 30, 36). Regardless of whether the experimental group was PD-1/PD-L1 plus chemotherapy or PD-1/PD-L1 inhibitor, the incidence risk of hepatitis in the PD-1/PD-L1 related experimental group was higher than that of the chemotherapy control group (RR = 5.52, 95% CI: [1.43, 21.38], *I*^2^ = 0%, *Z* = 2.47 (*P* = 0.01), Figure 4A; RR = 2.21, 95% CI: [1.21, 4.06], *I*^2^ = 0%, *Z* = 2.56 (*P* = 0.01), Figure 4B) (12, 14–18, 20, 21, 30, 36). Similar incidence trend happened in another two groups, which the experimental group was Nivolumab plus Ipilimumab and the control group was Nivolumab or Ipilimumab separately (RR = 6.85, 95% CI: [1.26, 37.19], *I*^2^ = 0%, *Z* = 2.23 (*P* = 0.03), Figure 4D; RR = 11.00, 95% CI: [1.42, 84.95], *I*^2^ = 0%, *Z* = 2.30 (*P* = 0.02), Figure 4E). No statistical significant results were seen in Figure 4C, which the control group was placebo (RR = 4.34, 95% CI: [0.75, 24.97], *I*^2^ = 0%, *Z* = 1.64 (*P* = 0.10) (22, 23). The corresponding funnel plots of RR were summarized in Supplemental Figure 4. No obvious heterogeneity was found among all groups (*I*^2^ = 0%).

### Incidence Risk of Pancreatitis

**Figure 5:**
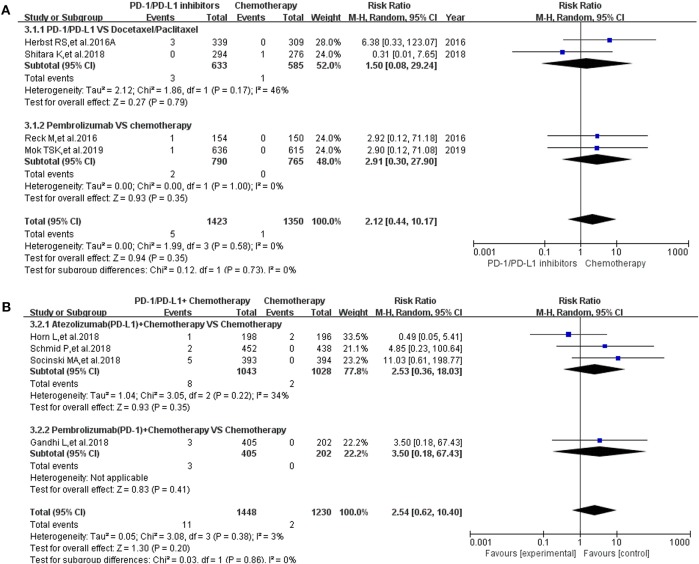
Forest plots for the risk ratio of pancreatitis for all grade. **(A)** Forest plots for the risk ratio of treatment related pancreatitis (PD-1/PD-L1 vs. Docetaxel/Paclitaxel or Chemotherapy). Subgroup analysis was performed according to the composition of chemotherapy in the control group. **(B)** Forest plots for the risk ratio of treatment related pancreatitis (PD-1/PD-L1 + Chemotherapy vs. Chemotherapy). Subgroup analysis was performed based on the drug type (PD-1 or PD-L1) of the experimental group.

**Figure 6:**
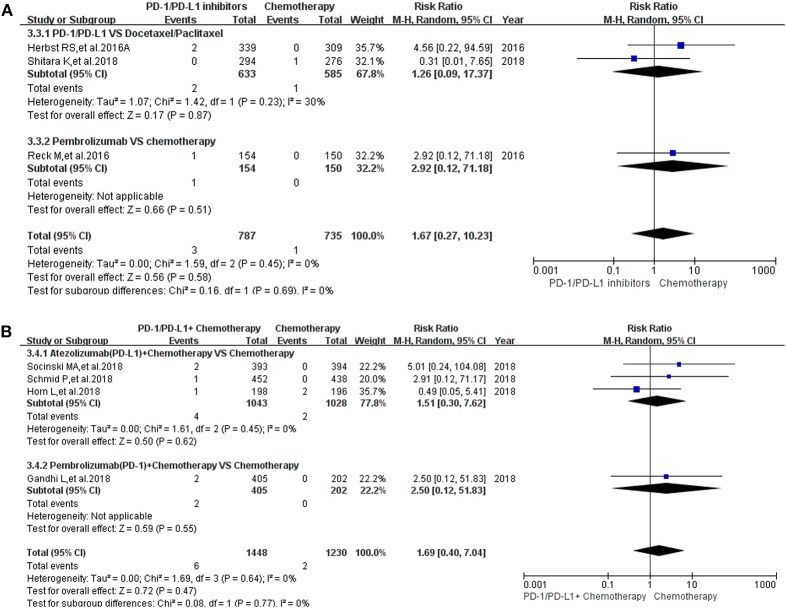
Forest plots for the risk ratio of pancreatitis for grade 3–5. **(A)** Forest plots for the risk ratio of treatment related pancreatitis (PD-1/PD-L1 vs. Docetaxel/Paclitaxel or Chemotherapy). Subgroup analysis was performed according to the composition of chemotherapy in the control group. **(B)** Forest plots for the risk ratio of treatment related pancreatitis (PD-1/PD-L1 + Chemotherapy vs. Chemotherapy). Subgroup analysis was performed based on the drug type (PD-1 or PD-L1) of the experimental group.

Pancreatitis was reported in 10 enrolled clinical trials (12, 15–17, 20, 22, 27, 30, 31, 36). Among them, 8 clinical trials with the information of all grade pancreatitis were taken into account for the final meta-analysis (12, 15–17, 20, 27, 30, 36). Regardless of whether the experimental group was PD-1/PD-L1 plus chemotherapy or PD-1/PD-L1 inhibitor, the incidence risk of pancreatitis in the PD-1/PD-L1 related experimental group was of no statistical significance (RR = 2.12, 95% CI: [0.44, 10.17], *I*^2^ = 0%, *Z* = 0.94 (*P* = 0.35), Figure 5A; RR = 2.54, 95% CI: [0.62, 10.40], *I*^2^ = 3%, *Z* = 1.30 (*P* = 0.20), Figure 5B) (12, 15–17, 20, 27, 30, 36). Similar analysis results of grade 3–5 pancreatitis could also be seen in Figure 6 (15–17, 20, 27, 30, 36). The corresponding funnel plots of RR were summarized in Supplemental Figures 5, 6. No obvious heterogeneity was found among all groups.

## Discussion

The hallmarks of cancer involves active evasion by cancer cells from attack and elimination by immune cells; this capability highlights the dichotomous roles of an immune system that both antagonizes and enhances tumor development and progression (43, 44). Therefore, cancer can also be defined as an immune-related disease (43, 44). In the field of anti-tumor therapy, immunosuppressants had been widely used as a new anti-tumor therapy in clinical practice, and had achieved gratifying clinical effects (11–36). With the development of anti-tumor immunosuppressants, a number of immune-related side effects had been reported, and immune-related digestive system inflammation was one of them (11–36). PD-1/PD-L1 inhibitor is the most commonly used anti-tumor immunosuppressant in clinical practice, and it is also one of the most common immunosuppressive drugs for causing immune gastrointestinal inflammatory diseases among cancer patients (7–36). To investigate the relationship between incidence risk of immune-related digestive system inflammation and PD-1/PD-L1 inhibitors, we performed this meta-analysis.

After screening and eligibility assessment, a total of 26 clinical trials involving 16,409 patients were enrolled for the final meta-analysis (11–36). All enrolled clinical trials were considered to be of higher good quality, when study quality and risk of bias among enrolled studies were evaluated by Newcastle-Ottawa scale (42). The evaluation results of bias was shown in Supplemental Figure 8 (42). Therefore, the analysis results based on the data from the above included trials had a high degree of credibility. However, due to the small number of studies included in the individual groups, sufficient subgroup analysis was not performed, which is also a shortcoming of this study.

The control group was chemotherapy, whether the experimental group was PD-1/PD-L1 inhibitor or PD-1/PD-L1 inhibitor plus chemotherapy, the results suggested that the use of PD-1/PD-L1 inhibitors increased the incidence risk of colitis (Figures 1A,B, 2A,B) (12, 14–20, 26–28, 30, 32–34, 36). Statistical significant analysis results could be seen in 3 groups (Figures 1A,B, 2A) (12, 15–20, 26, 27, 30, 32, 33, 36). When Nivolumab (PD-1) was compared with Ipilimumab (anti-CTLA4), regardless of the composition of the experimental and control groups, the results showed that Nivolumab had a lower incidence risk of colitis than Ipilimumab (Figures 1C,E,F, 2C,E,F) (13, 14, 25, 29, 31, 35), which 4 analysis results of them were shown with statistical differences (Figures 1E,F, 2E,F) (13, 24, 25, 29, 35). The similar incidence trend could also be seen when Nivolumab was compared to placebo (Figures 1D, 2D) (22, 23). Obvious heterogeneity was only found in Figure 1F (*I*^2^ = 68%) (13, 24, 25, 35). After comparing Figure 1F with Figure 2F, we found that the heterogeneity might come from the enrolled trial (25).

16 clinical trials with the information of all grade hepatitis were taken into account for meta-analysis (12–18, 20–25, 30, 31, 36). When PD-1/PD-L1 inhibitors plus chemotherapy was compared with chemotherapy, the analysis result of incidence risk of all grade hepatitis was considered to be no significant (RR = 1.89, 95% CI: [0.86, 4.19], *I*^2^ = 50%, *Z* = 1.58 (*P* = 0.11); Figure 3B) (15–18, 36). Obvious significant difference was seen when they were used for evaluating the incidence risk of grade 3–5 hepatitis (RR = 2.21, 95% CI: [1.21, 4.06], *I*^2^ = 0%, *Z* = 2.56 (*P* = 0.01), Figure 4B) (15–18, 36). Similar incidence trend could also be seen when PD-1 inhibitor was compared to Ipilimumab (Figures 3F, 4F) (13, 14, 25). Moderate heterogeneity was only found in Figure 3B (*I*^2^ = 50%) (15– 18, 36). By comparing the results of the analysis in Figures 3B, 4B, we still could not determine the source of heterogeneity. Therefore, we concluded that heterogeneity might originate from the data themselves. Publication bias, checked by Harbord's test (37), was not found in the form of funnel plots (Supplemental Figures 1–6).

Through a comprehensive analysis, we found that the effect of PD-1/PD-L1 inhibitors on the risk of immune related hepatitis was roughly the same as that for immune related colitis (Figures 3, 4), while no significant statistical results were seen for the analysis of immune related pancreatitis (Figures 5, 6) (12, 15–17, 20, 22, 27, 30, 31, 36). This might be related to the low incidence of immune-related pancreatitis and the small number of patients included in the study (Figures 5, 6) (12, 15–17, 20, 22, 27, 30, 31, 36).

For drug-induced immune related digestive system inflammation of grade 3–5, stopping the use of the corresponding induced drug remained the primary treatment option (11–13, 15–20, 22–24, 26, 27, 29–36). However, due to the particularity of the tumor patient population, in clinical work, the discontinuation anti-tumor treatment should be carefully considered to prevent the sudden stop of all anti-tumor drugs leading to rapid tumor progression, endangering the lives of patients (11–36). Through the above analysis, we found that PD-1/PD-L1 inhibitors can increase the risk of developing colitis and hepatitis. Therefore, when we encountered the need to stop anti-tumor therapy to alleviate severe immune related digestive system inflammation, it was preferred to stop the PD-1/PD-L1 inhibitor (11–36). This had an important guiding significance for us to determine the cause of immune related digestive system inflammation and the adjustment of clinical treatment.

## Conclusions

The use of PD-1/PD-L1 inhibitors increased the incidence risk of immune-related colitis and hepatitis, but this potential to increase the incidence risk of the disease was weaker than Ipilimumab.

## Data Availability Statement

All datasets analyzed for this study are included in the article/Supplementary Material.

## Author Contributions

YT had full access to all data in the study and all authors had final responsibility for the decision to submit for publication. ZZ, XY, DL, LZ, and YT had the full data of the paper. ZL, SZ, YM, CJ, and YZ were responsible for the collection of clinical data. ZZ helped to gather the online data and write the report.

### Conflict of Interest

The authors declare that the research was conducted in the absence of any commercial or financial relationships that could be construed as a potential conflict of interest.

## Supplementary Material

Supplemental Figure 1 Funnel plots for the risk ratio of colitis for all grade. **(A)** Funnel plots for the risk ratio of treatment related colitis (PD-1/PD-L1 vs. Docetaxel/Paclitaxel or Chemotherapy). Subgroup analysis was performed according to the composition of chemotherapy in the control group. **(B)** Funnel plots for the risk ratio of treatment related colitis (PD-1/PD-L1 + Chemotherapy vs. Chemotherapy). Subgroup analysis was performed based on the drug type (PD-1 or PD-L1) of the experimental group. **(C)** Funnel plots for the risk ratio of treatment related colitis (Nivolumab + Ipilimumab vs. Ipilimumab). **(D)** Funnel plots for the risk ratio of treatment related colitis (PD-1 vs. Placebo). **(E)** Funnel plots for the risk ratio of treatment related colitis (Nivolumab vs. Nivolumab + Ipilimumab). Subgroup analysis was performed according to the tumor type. **(F)** Funnel plots for the risk ratio of treatment related colitis (Nivolumab vs. Ipilimumab).

Supplemental Figure 2 Funnel plots for the risk ratio of colitis for grade 3–5. **(A)** Funnel plots for the risk ratio of treatment related colitis (PD-1/PD-L1 vs. Docetaxel/Paclitaxel or Chemotherapy). Subgroup analysis was performed according to the composition of chemotherapy in the control group. **(B)** Funnel plots for the risk ratio of treatment related colitis (PD-1/PD-L1 + Chemotherapy vs. Chemotherapy). Subgroup analysis was performed based on the drug type (PD-1 or PD-L1) of the experimental group. **(C)** Funnel plots for the risk ratio of treatment related colitis (Nivolumab + Ipilimumab vs. Ipilimumab). **(D)** Funnel plots for the risk ratio of treatment related colitis (PD-1 vs. Placebo). **(E)** Funnel plots for the risk ratio of treatment related colitis (Nivolumab vs. Nivolumab + Ipilimumab). Subgroup analysis was performed according to the tumor type. **(F)** Funnel plots for the risk ratio of treatment related colitis (Nivolumab vs. Ipilimumab).

Supplemental Figure 3 Funnel plots for the risk ratio of hepatitis for all grade. **(A)** Funnel plots for the risk ratio of treatment related hepatitis (PD-1/PD-L1 vs. Docetaxel/Paclitaxel or Chemotherapy). Subgroup analysis was performed according to the composition of chemotherapy in the control group. **(B)** Funnel plots for the risk ratio of treatment related hepatitis (PD-1/PD-L1 + Chemotherapy vs. Chemotherapy). Subgroup analysis was performed based on the drug type (PD-1 or PD-L1) of the experimental group. **(C)** Funnel plots for the risk ratio of treatment related hepatitis (PD-1 vs. Placebo). **(D)** Funnel plots for the risk ratio of treatment related hepatitis (Nivolumab + Ipilimumab vs. Ipilimumab). **(E)** Funnel plots for the risk ratio of treatment related hepatitis (Nivolumab + Ipilimumab vs. Nivolumab). **(F)** Funnel plots for the risk ratio of treatment related hepatitis (Nivolumab vs. Ipilimumab).

Supplemental Figure 4 Funnel plots for the risk ratio of hepatitis for grade 3–5. **(A)** Funnel plots for the risk ratio of treatment related hepatitis (PD-1/PD-L1 vs. Docetaxel/Paclitaxel or Chemotherapy). Subgroup analysis was performed according to the composition of chemotherapy in the control group. **(B)** Funnel plots for the risk ratio of treatment related hepatitis (PD-1/PD-L1 + Chemotherapy vs. Chemotherapy). Subgroup analysis was performed based on the drug type (PD-1 or PD-L1) of the experimental group. **(C)** Funnel plots for the risk ratio of treatment related hepatitis (PD-1 vs. Placebo). **(D)** Funnel plots for the risk ratio of treatment related hepatitis (Nivolumab + Ipilimumab vs. Ipilimumab). **(E)** Funnel plots for the risk ratio of treatment related hepatitis (Nivolumab + Ipilimumab vs. Nivolumab).

Supplemental Figure 5 Funnel plots for the risk ratio of pancreatitis for all grade. **(A)** Funnel plots for the risk ratio of treatment related pancreatitis (PD-1/PD-L1 vs. Docetaxel/Paclitaxel or Chemotherapy). Subgroup analysis was performed according to the composition of chemotherapy in the control group. **(B)** Funnel plots for the risk ratio of treatment related pancreatitis (PD-1/PD-L1 + Chemotherapy vs. Chemotherapy). Subgroup analysis was performed based on the drug type (PD-1 or PD-L1) of the experimental group.

Supplemental Figure 6 Funnel plots for the risk ratio of pancreatitis for grade 3–5. **(A)** Funnel plots for the risk ratio of treatment related pancreatitis (PD-1/PD-L1 vs. Docetaxel/Paclitaxel or Chemotherapy). Subgroup analysis was performed according to the composition of chemotherapy in the control group. **(B)** Funnel plots for the risk ratio of treatment related pancreatitis (PD-1/PD-L1 + Chemotherapy vs. Chemotherapy). Subgroup analysis was performed based on the drug type (PD-1 or PD-L1) of the experimental group.

Supplemental Figure 7 PRISMA flow diagram.

Supplemental Figure 8 Risk of bias summary: review authors' judgments about each risk of bias item for each included study.

## Abbreviations

PRISMA: Preferred Reporting Items for Systematic Reviews and Meta-Analyses
PD-1: programmed cell death-1
PD-L1: programmed cell death ligand 1
HR: hazard ratios
RR: risk ratio
CI: confidence interval
RE: random effect
FE: fixed effect.
