# Supplementary figures and images for: The Risk Ratio of Immune-Related Colitis, Hepatitis, and Pancreatitis in Patients With Solid Tumors Caused by PD-1/PD-L1 Inhibitors: A Systematic Review and Meta-Analysis
Source: Front Oncol. 2020 Feb 28;10:261. doi: 10.3389/fonc.2020.00261 (PMC7059308; doi:10.3389/fonc.2020.00261)

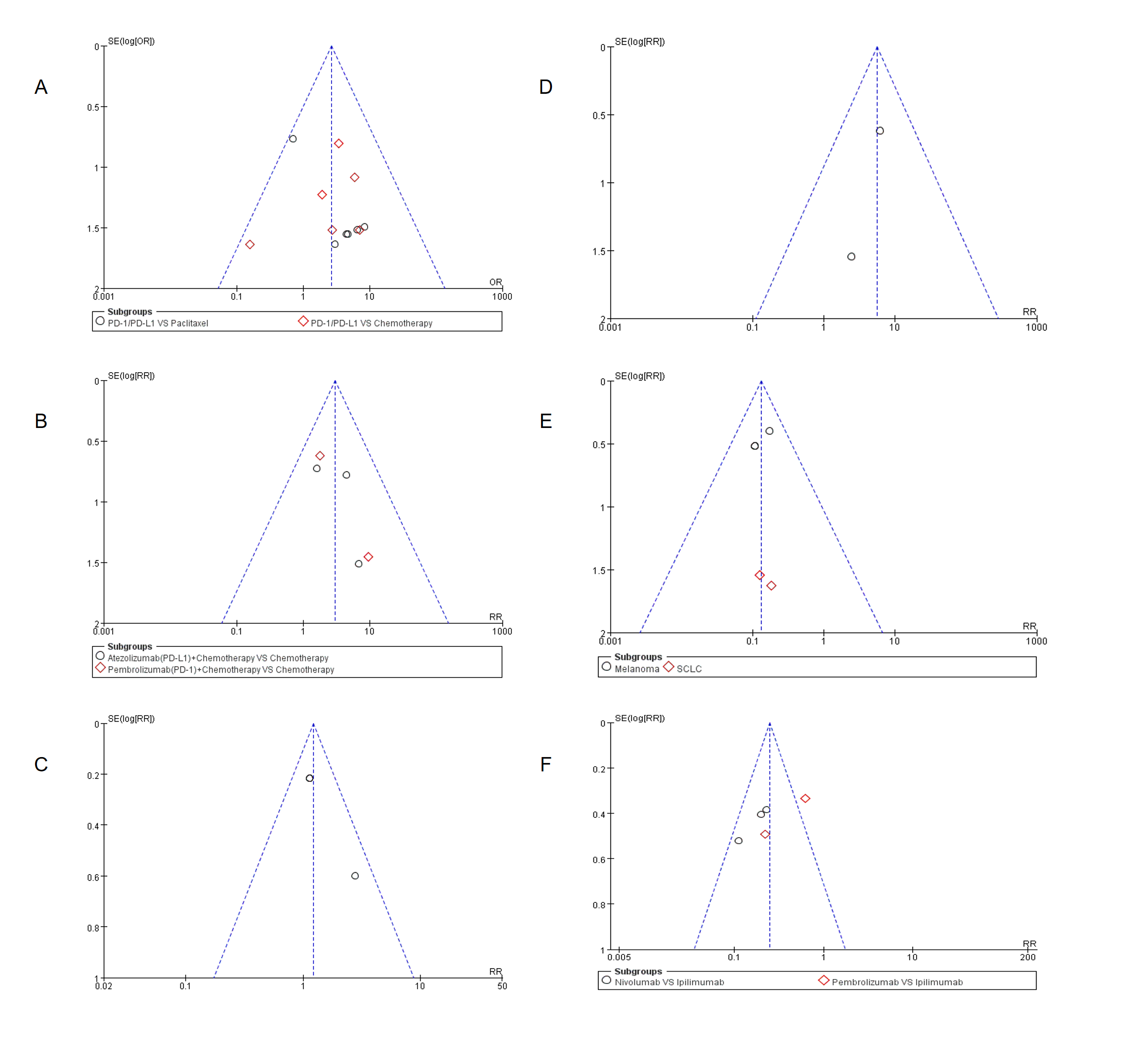

Supplement: Supplemental Figure 1 — Funnel plots for the risk ratio of colitis for all grade. (A) Funnel plots for the risk ratio of treatment related colitis (PD-1/PD-L1 vs. Docetaxel/Paclitaxel or Chemotherapy). Subgroup analysis was performed according to the composition of chemotherapy in the control group. (B) Funnel plots for the risk ratio of treatment related colitis (PD-1/PD-L1 + Chemotherapy vs. Chemotherapy). Subgroup analysis was performed based on the drug type (PD-1 or PD-L1) of the experimental group. (C) Funnel plots for the risk ratio of treatment related colitis (Nivolumab + Ipilimumab vs. Ipilimumab). (D) Funnel plots for the risk ratio of treatment related colitis (PD-1 vs. Placebo). (E) Funnel plots for the risk ratio of treatment related colitis (Nivolumab vs. Nivolumab + Ipilimumab). Subgroup analysis was performed according to the tumor type. (F) Funnel plots for the risk ratio of treatment related colitis (Nivolumab vs. Ipilimumab). [file Image_1.TIF]

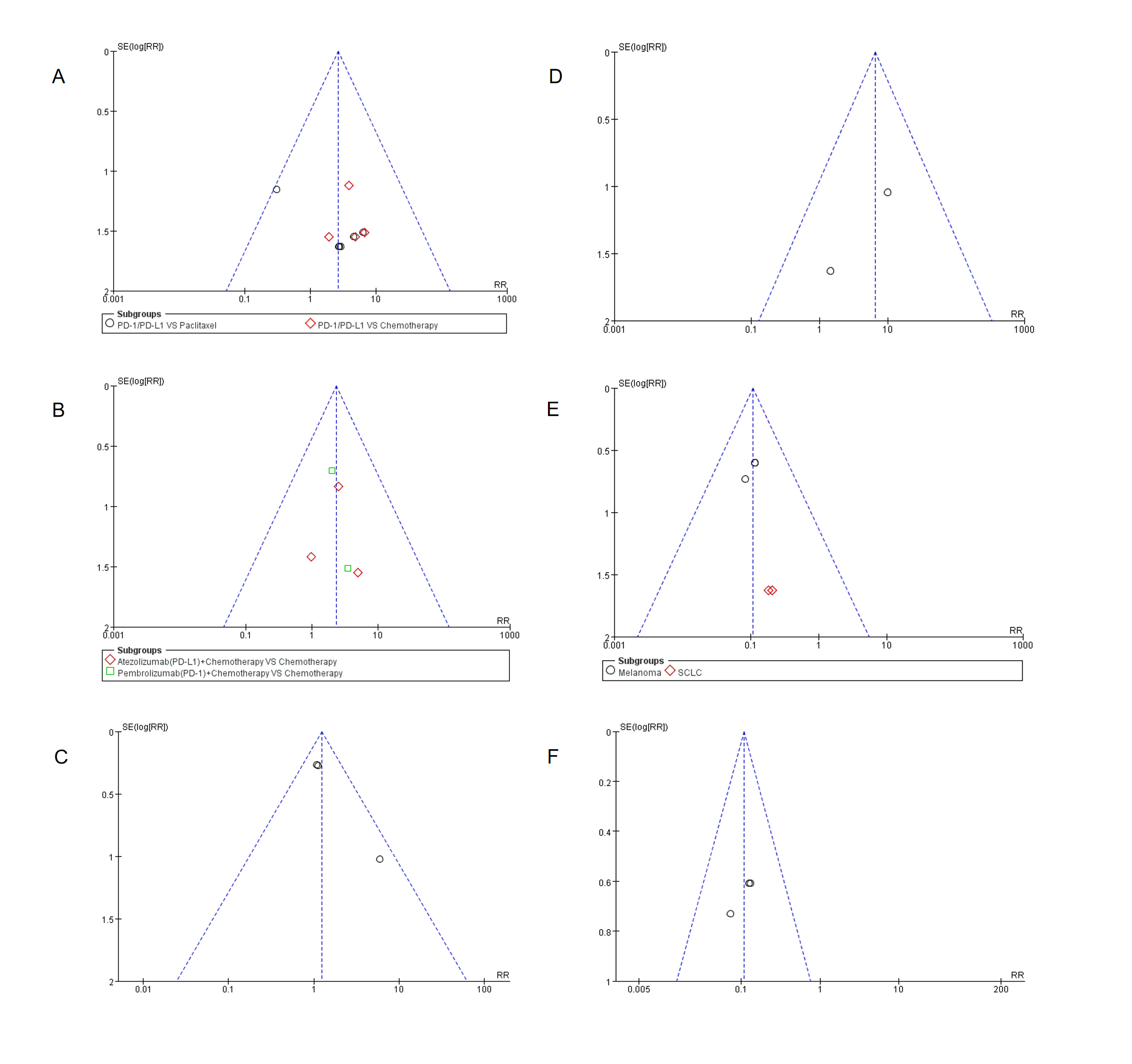

Supplement: Supplemental Figure 2 — Funnel plots for the risk ratio of colitis for grade 3–5. (A) Funnel plots for the risk ratio of treatment related colitis (PD-1/PD-L1 vs. Docetaxel/Paclitaxel or Chemotherapy). Subgroup analysis was performed according to the composition of chemotherapy in the control group. (B) Funnel plots for the risk ratio of treatment related colitis (PD-1/PD-L1 + Chemotherapy vs. Chemotherapy). Subgroup analysis was performed based on the drug type (PD-1 or PD-L1) of the experimental group. (C) Funnel plots for the risk ratio of treatment related colitis (Nivolumab + Ipilimumab vs. Ipilimumab). (D) Funnel plots for the risk ratio of treatment related colitis (PD-1 vs. Placebo). (E) Funnel plots for the risk ratio of treatment related colitis (Nivolumab vs. Nivolumab + Ipilimumab). Subgroup analysis was performed according to the tumor type. (F) Funnel plots for the risk ratio of treatment related colitis (Nivolumab vs. Ipilimumab). [file Image_2.TIF]

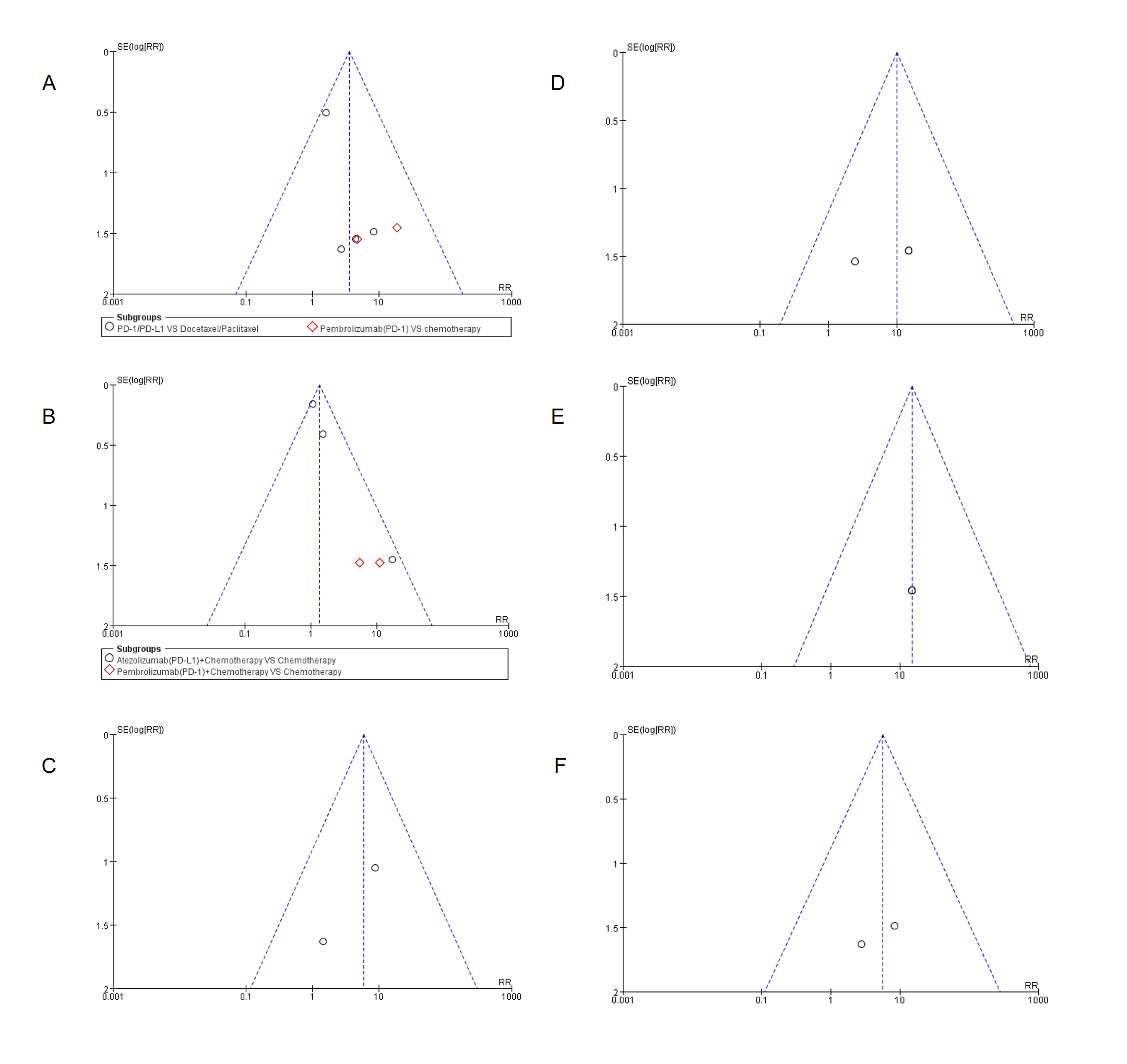

Supplement: Supplemental Figure 3 — Funnel plots for the risk ratio of hepatitis for all grade. (A) Funnel plots for the risk ratio of treatment related hepatitis (PD-1/PD-L1 vs. Docetaxel/Paclitaxel or Chemotherapy). Subgroup analysis was performed according to the composition of chemotherapy in the control group. (B) Funnel plots for the risk ratio of treatment related hepatitis (PD-1/PD-L1 + Chemotherapy vs. Chemotherapy). Subgroup analysis was performed based on the drug type (PD-1 or PD-L1) of the experimental group. (C) Funnel plots for the risk ratio of treatment related hepatitis (PD-1 vs. Placebo). (D) Funnel plots for the risk ratio of treatment related hepatitis (Nivolumab + Ipilimumab vs. Ipilimumab). (E) Funnel plots for the risk ratio of treatment related hepatitis (Nivolumab + Ipilimumab vs. Nivolumab). (F) Funnel plots for the risk ratio of treatment related hepatitis (Nivolumab vs. Ipilimumab). [file Image_3.TIF]

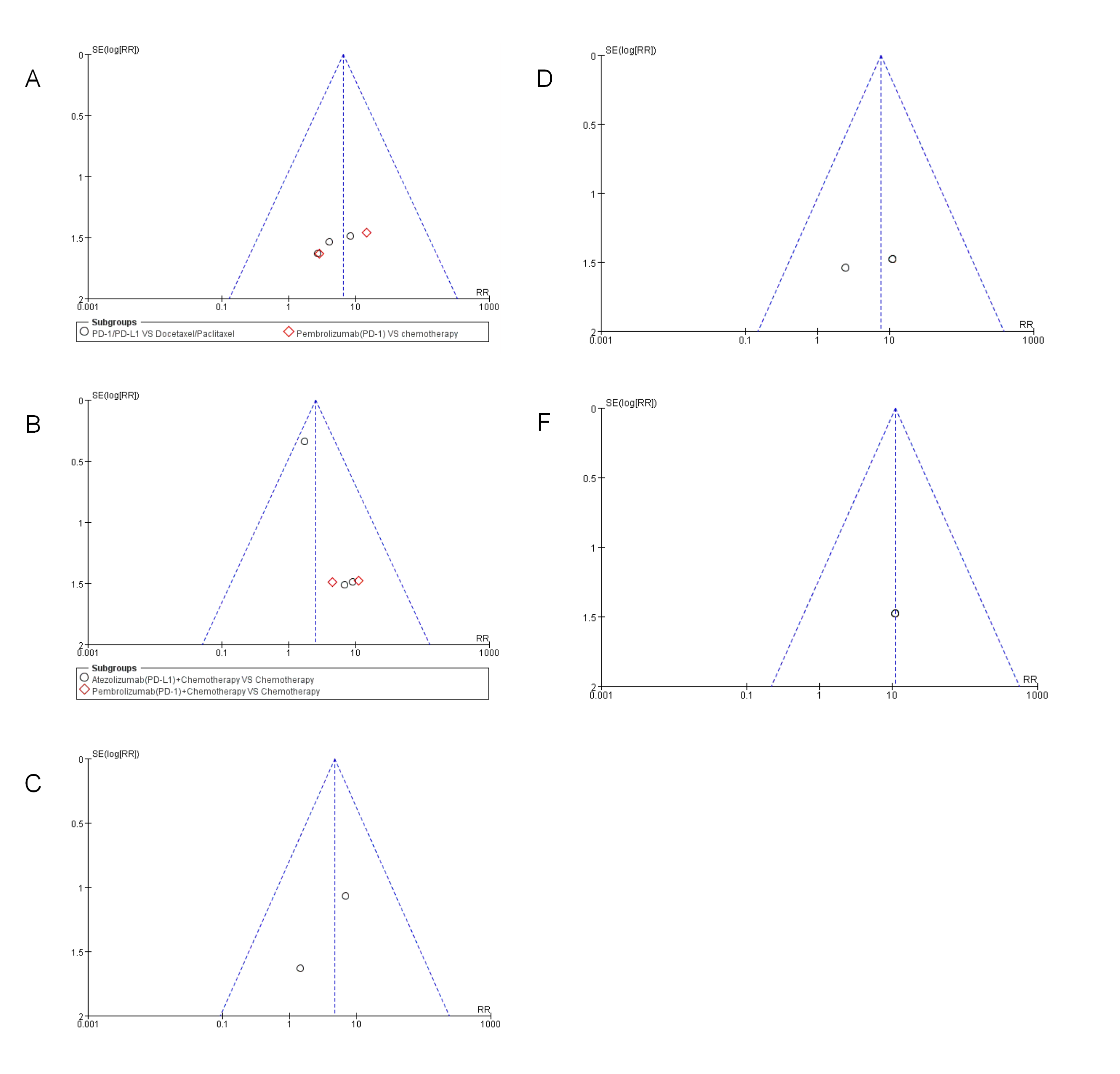

Supplement: Supplemental Figure 4 — Funnel plots for the risk ratio of hepatitis for grade 3–5. (A) Funnel plots for the risk ratio of treatment related hepatitis (PD-1/PD-L1 vs. Docetaxel/Paclitaxel or Chemotherapy). Subgroup analysis was performed according to the composition of chemotherapy in the control group. (B) Funnel plots for the risk ratio of treatment related hepatitis (PD-1/PD-L1 + Chemotherapy vs. Chemotherapy). Subgroup analysis was performed based on the drug type (PD-1 or PD-L1) of the experimental group. (C) Funnel plots for the risk ratio of treatment related hepatitis (PD-1 vs. Placebo). (D) Funnel plots for the risk ratio of treatment related hepatitis (Nivolumab + Ipilimumab vs. Ipilimumab). (E) Funnel plots for the risk ratio of treatment related hepatitis (Nivolumab + Ipilimumab vs. Nivolumab). [file Image_4.TIF]

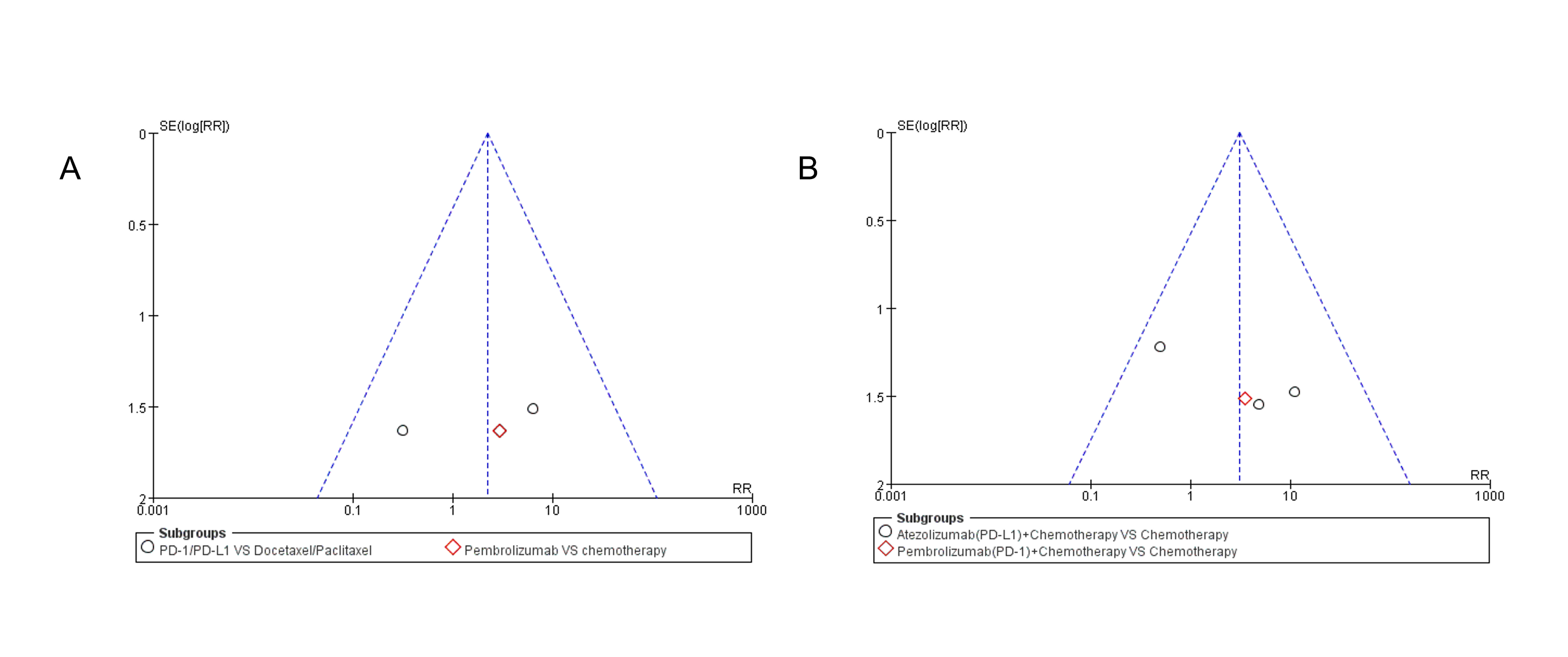

Supplement: Supplemental Figure 5 — Funnel plots for the risk ratio of pancreatitis for all grade. (A) Funnel plots for the risk ratio of treatment related pancreatitis (PD-1/PD-L1 vs. Docetaxel/Paclitaxel or Chemotherapy). Subgroup analysis was performed according to the composition of chemotherapy in the control group. (B) Funnel plots for the risk ratio of treatment related pancreatitis (PD-1/PD-L1 + Chemotherapy vs. Chemotherapy). Subgroup analysis was performed based on the drug type (PD-1 or PD-L1) of the experimental group. [file Image_5.TIF]

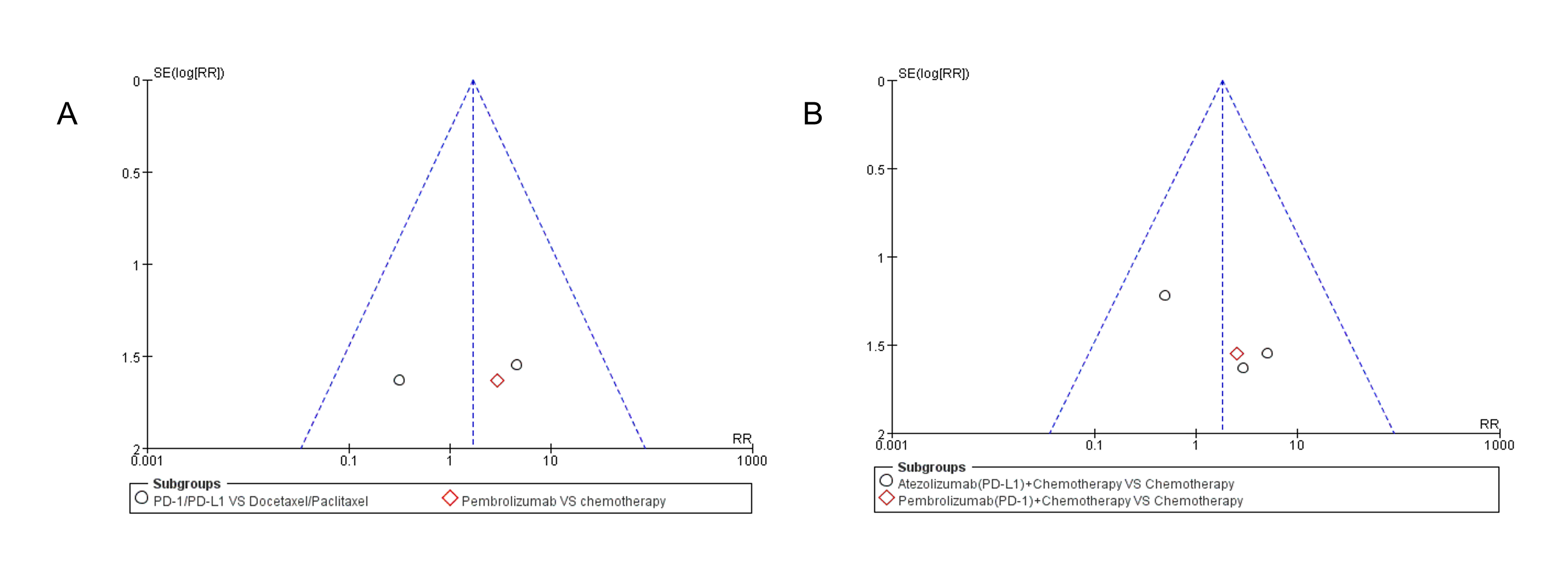

Supplement: Supplemental Figure 6 — Funnel plots for the risk ratio of pancreatitis for grade 3–5. (A) Funnel plots for the risk ratio of treatment related pancreatitis (PD-1/PD-L1 vs. Docetaxel/Paclitaxel or Chemotherapy). Subgroup analysis was performed according to the composition of chemotherapy in the control group. (B) Funnel plots for the risk ratio of treatment related pancreatitis (PD-1/PD-L1 + Chemotherapy vs. Chemotherapy). Subgroup analysis was performed based on the drug type (PD-1 or PD-L1) of the experimental group. [file Image_6.TIF]

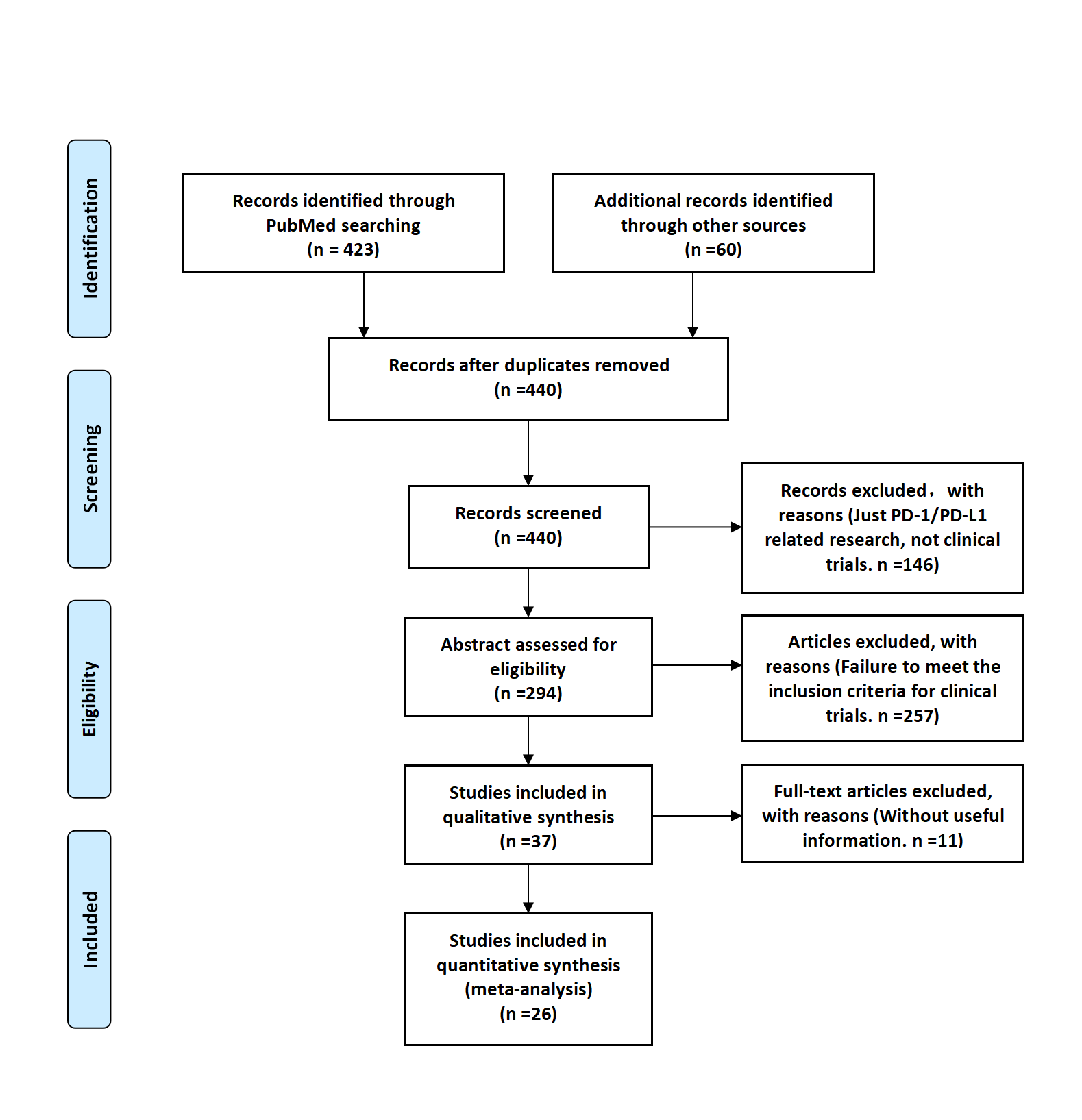

Supplement: Supplemental Figure 7 — PRISMA flow diagram. [file Image_7.TIF]

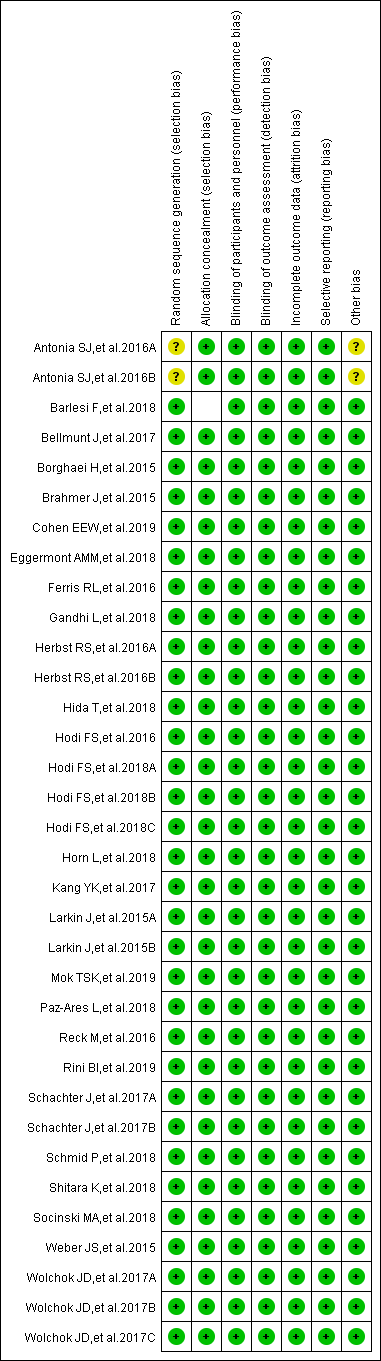

Supplement: Supplemental Figure 8 — Risk of bias summary: review authors' judgments about each risk of bias item for each included study. [file Image_8.PNG]
